# Kinematics of swimming and thrust production during powerstroking bouts of the swim frenzy in green turtle hatchlings

**DOI:** 10.1242/bio.20149480

**Published:** 2014-09-04

**Authors:** David T. Booth

**Affiliations:** School of Biological Sciences, The University of Queensland, St Lucia, QLD 4072, Australia

**Keywords:** Swimming, Kinematics, Thrust, Hatchlings, Sea turtle, Green turtle, Frenzy swim

## Abstract

Hatchling sea turtles emerge from nests, crawl down the beach and enter the sea where they typically enter a stereotypical hyperactive swimming frenzy. During this swim the front flippers are moved up and down in a flapping motion and are the primary source of thrust production. I used high-speed video linked with simultaneous measurement of thrust production in tethered hatchlings, along with high-speed video of free swimming hatchlings swimming at different water speeds in a swim flume to investigate the links between kinematics of front flipper movement, thrust production and swimming speed. In particular I tested the hypotheses that (1) increased swimming speed is achieved through an increased stroke rate; (2) force produced per stroke is proportional to stroke amplitude, (3) that forward thrust is produced during both the down and up phases of stroking; and (4) that peak thrust is produced towards the end of the downstroke cycle. Front flipper stroke rate was independent of water speed refuting the hypothesis that swimming speed is increased by increasing stroke rate. Instead differences in swimming speed were caused by a combination of varying flipper amplitude and the proportion of time spent powerstroking. Peak thrust produced per stroke varied within and between bouts of powerstroking, and these peaks in thrust were correlated with both flipper amplitude and flipper angular momentum during the downstroke supporting the hypothesis that stroke force is a function of stroke amplitude. Two distinct thrust production patterns were identified, monophasic in which a single peak in thrust was recorded during the later stages of the downstroke, and biphasic in which a small peak in thrust was recorded at the very end of the upstroke and this followed by a large peak in thrust during the later stages of the downstroke. The biphasic cycle occurs in ∼20% of hatchlings when they first started swimming, but disappeared after one to two hours of swimming. The hypothesis that forward thrust is produced during both the up and down stroke was only supported relatively rarely in hatchlings that exhibited the diphasic cycle, the majority of time forward thrust was only produced during the downstroke phase. The hypothesis that peak forward thrust is produced during the end of the downstroke was supported in both the monophasic and biphasic thrust producing stroke cycles.

## INTRODUCTION

Hatchling sea turtles emerge from their nests scramble down the beach and enter the sea where they swim off-shore and enter a pelagic stage of their life-history (Spotila, 2004). The near-shore waters are inhabited by high densities of turtle predators and, as a consequence, high predation rates are typically experienced within the first few minutes to hours of entering the water (Gyuris, 1994; Gyuris, 2000; Pilcher et al., 2000; Whelan and Wyneken, 2007). Thus, by minimizing the time spent in near-shore waters, hatchlings can maximize their chances of survival. Hatchling green (*Chelonia mydas* Linnaeus), loggerhead (*Caretta caretta* Linnaeus) and leatherback (*Dermochelys coriacea* Linnaeus) turtles minimize the time they spend near to shore by immediately engaging in a hyperactive (frenzy) swimming phase that lasts up to 24 h immediately after they enter the water (Wyneken and Salmon, 1992). Similarly, flatback (*Natator depressus* Garman) hatchlings also enter a swim frenzy immediately upon entering the water (Pereira et al., 2011), but this can last up to 4 days (Salmon et al., 2009). Swimming is achieved by ‘powerstroking’ bouts lasting 5–10 s in which the front flippers are moved in an up and down flying motion which generate forward thrust (Carr and Ogren, 1960; Davenport et al., 1984; Salmon and Wyneken, 1987; Wyneken, 1997). These powerstroking bouts are typically separated by brief 2–5 s periods of ‘dog paddling’ when the head is raised to take a breath and the gait switches from a front flippers only movement to one in which diagonally opposite flippers move together (Salmon and Wyneken, 1987; Wyneken, 1997). The thrust produced during powerstroking greatly exceeds that produced during dog paddling, and the proportion of time spent powerstroking remains relatively constant in loggerhead hatchlings, but decreases slightly in green hatchlings and decreases greatly in flatback hatchlings as the swimming frenzy progresses (Pereira et al., 2011). Stroke rate during a powerstroke bout, maximum thrust produced per stroke and, consequently, mean thrust produced decreases during the first eight hours of swimming in green, loggerhead and flatback hatchlings, with the greatest reduction occurring within the first two hours of entering the water (Pereira et al., 2011).

High speed cinematography or video has been used to describe the kinematics of swimming in hatchling and post-hatching green and loggerhead turtles (Davenport et al., 1984; Wyneken, 1997; Becking et al., 2004; Rivera et al., 2009; Rivera et al., 2011; Rivera et al., 2012), and thrust production has also been directly measured in two of these studies (Davenport et al., 1984; Becking et al., 2004). From these studies it is known that during powerstroking the downstroke is faster than the upstroke, that forward thrust is generated during both the downstroke and upstroke (although (Becking et al., 2004) found that in hatchlings forward thrust is only produced on the downstroke), and that the thrust produced during the downstroke is greater than the thrust produced during the upstroke. However, only one study has examined the kinematics and thrust production directly in newly emerged hatchlings and this study was published in the form of an abstract and thus lacks detail (Becking et al., 2004). The current study used simultaneous high speed digital videography and measurement of thrust production in newly emerged green turtle hatchlings to further describe the kinematics of swimming. In particular I tested the hypotheses that (1) increased swimming speed is achieved through an increased stroke rate; (2) force produced per stroke is proportional to stroke amplitude, (3) what forward thrust is produced during both the down and up phases of the stroke cycle; and (4) that peak thrust is produced towards the end of the downstroke. Two types of experiments were conducted, tethered hatchling experiments to investigate relationships between force production and flipper movement, and swim flume experiments to investigate the relationship between stroke rate and swimming speed. In flume experiments water speeds between 0.2 and 0.5 m/s were used as this covers most of the range of mean swimming speeds reported for green turtle hatchlings during their frenzy swim in nature (0.23 to 0.59 m/s) (Frick, 1976; Pilcher et al., 2000; Wyneken, 1997; Okuyama et al., 2009).

## RESULTS

### Flume swimming experiments

All hatchlings swum 3 to 10 cm below the water's surface while powerstroking and their flippers never broke the surface of the water during any of the analysed powerstroking bouts (supplementary material Movie 1). Six hatchlings were swum from each of four clutches (n  =  24), however only 12 hatchlings were able to maintain their position in water column at water speeds of 0.45 and 0.5 m/s (2 from clutch 1, 3 from clutch 2, 4 from clutch 3, and 3 from clutch 4) so only data from these 12 hatchlings were included in analysis. While dog paddling, hatchlings typically moved forward through the water flume at water speeds of 0.20, 0.25 and 0.30 m/s, remained stationary in the water flume at water speeds of 0.35 and 0.40 m/s and slipped backwards in the flume at water speeds of 0.45 and 0.50 m/s. While powerstroking hatchlings typically moved forward through the water flume at water speeds between 0.20 and 0.40 m/s, and typically remained stationary in the flume at water speeds of 0.45 and 0.50 m/s, but four hatchlings drifted backwards at 0.50 m/s. For each hatchling at each water speed, typically five different powerstroking bouts (at 0.20 and 0.25 m/s sometimes only 3 or 4 bouts could be analysed), each consisting of at least five consecutive powerstrokes were used for analysis. From these data, means were calculated for each hatchling at each water speed, and these means then used in repeated measures ANOVAs.

**Fig. 1.**
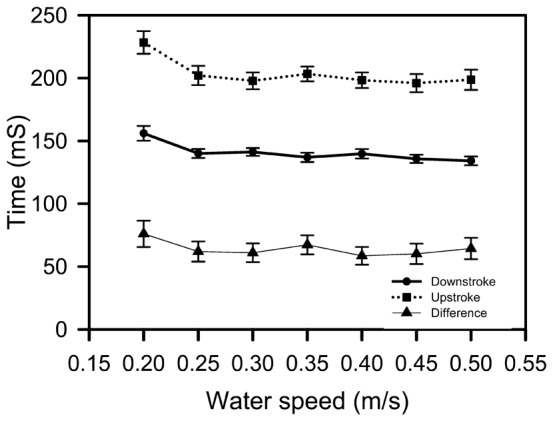
The relationship between water speed and the time taken for a downstroke, upstroke and the difference between downstroke and upstroke time for green turtle hatchlings in swimming flume trials (N  =  12, mass 24.9 ± 0.5 g, CL 50.0 ± 0.5 mm, CW 39.3 ± 0.4 mm).

Of the 1,937 power strokes analysed across all water speeds, downstrokes were on average 55 ms shorter than upstrokes with 88% of downstrokes being faster or equal to upstroke duration. Downstroke (F_(6,84)_  =  6.14, P < 0.001), upstroke (F_(6,84)_  =  6.82, P < 0.001) and the difference between downstroke and upstroke (F_(6,84)_  =  2.37, P  =  0.039) times varied with water speed with all three variables being similar at water speeds of 0.25 m/s and greater, but these were different to values recorded at 0.2 m/s (Fig. 1).

**Fig. 2.**
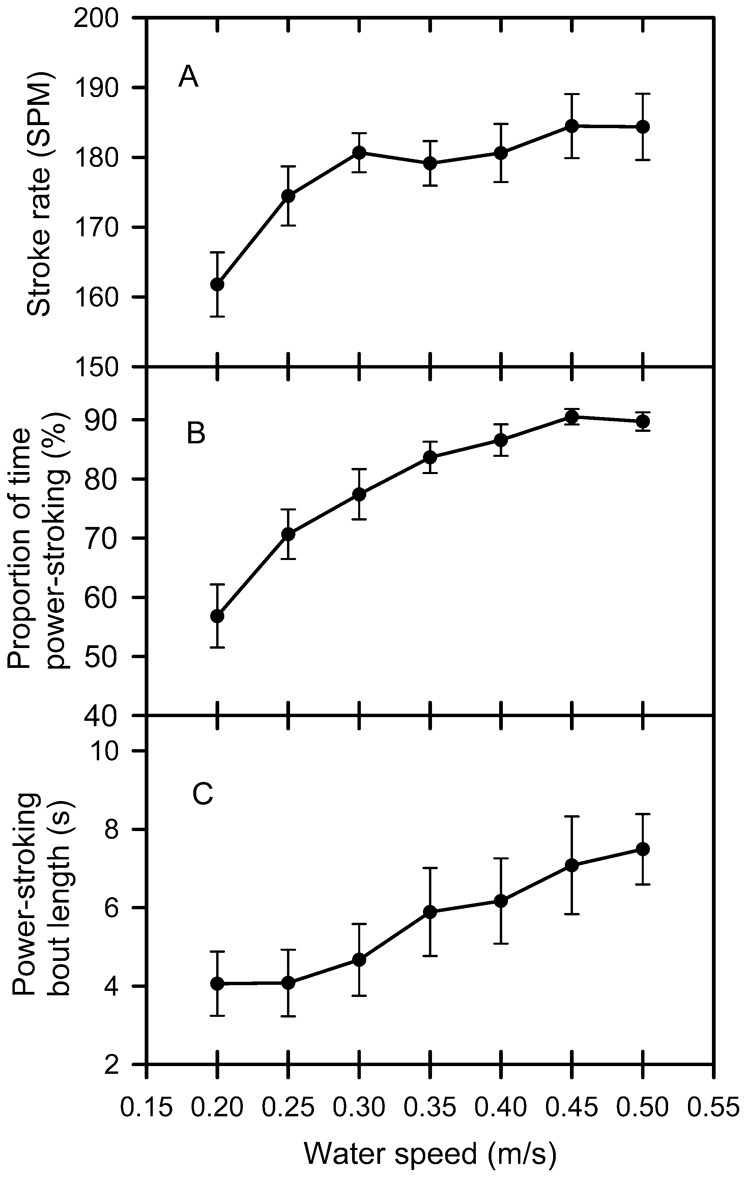
The relationship between (A) strokes rate during a powerstroking bout (stokes per minute SPM), (B) proportion of time spent powerstroking, and (C) the length of time an individual powerstroking bout and water speed for hatchling green turtles. Post-hoc analysis indicated that stroke rate was not significantly different at water speeds of 0.25–0.50 m/s, but was slower at 0.20 m/s. Post-hoc analysis indicated proportion of time spent powerstroking increased with water speed up to 0.45 m/s. Post-hoc analysis indicated powerstroking bout length increased at water speeds greater than 0.25 m/s.

For each hatchling at each water speed, the video was analysed for the proportion of time spent powerstroking by adding up the time of each powerstroking bout and dividing this value by the total time length of the video. These proportions were arcsin transformed before being analysed with repeated measures ANOVA. Powerstroking rate (calculated by dividing the number of strokes taken in a powerstroking bout by the time taken for that powerstroking bout) (F_(6,84)_  =  9.25, P < 0.001), the proportion of time spent powerstroking (F_(6,84)_  =  53.02, P < 0.001), and the length of time that a powerstroking bout lasted (F_(6,84)_  =  13.58, P < 0.0001) all increased in a non-linear manner with water speed (Fig. 2).

### Tethered swimming experiments

**Fig. 3.**
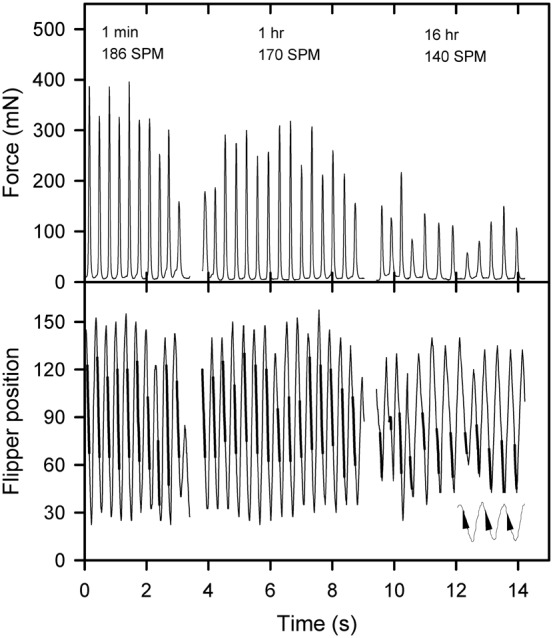
Traces of force production and mean front flipper tip position (in degrees) and stroke rate (strokes per minute SPM) during three different time intervals for a green turtle hatchling (25.6 g, CL 50.3 mm, CW 39.2 mm) exhibiting the monophasic force producing swimming style. Heavy lines in the flipper position trace indicate times when the thrust force was increasing. Insert in lower panel indicates the relative magnitude of the force applied during the downstroke, the thicker the line, the greater the force.

**Fig. 4.**
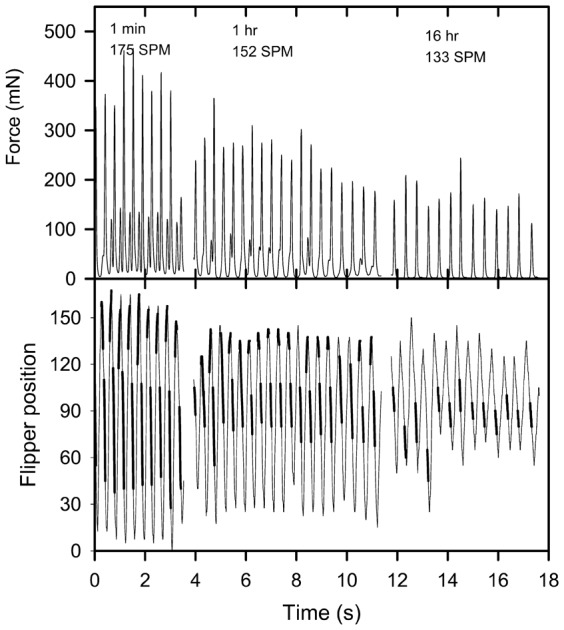
Traces of force production and mean front flipper tip position (in degrees) and stroke rate (strokes per minute SPM) during three different time intervals for a green turtle hatchling (28.6 g, CL 51.4 mm, CW 37.4 mm) exhibiting the biphasic force producing swimming style. Heavy lines in the flipper position trace indicate times when the thrust force was increasing.

All tethered hatchlings swam at or just below the water's surface and the tips of their flippers frequently broke the water's surface during powerstroking bouts. Power strokes involve extremely complicated 3-dimensional movement of the front flippers, including rotation of the blade angle, flexing of distal ends of flippers, anterior-dorsal bending and dorsal-ventral movement of flippers as well as the body pitching and heaving up and down within the water column (supplementary material Movie 2). The videos were not of sufficiently high resolution to comprehensively analyse this extremely complex flipper movement, but as a first, highly simplified analysis, the up and down flipper sweep angle was quantitatively assessed. All videos indicated similar up and down flipper movement, and because of the extremely laborious task of quantifying the flipper angles every 0.01 s during swimming only two typical hatchlings were quantitatively analysed at three separate time periods: within the first minute of being placed in the water, after one hour of swimming and after 16 hours of swimming (Figs 3, 4).

**Fig. 5.**
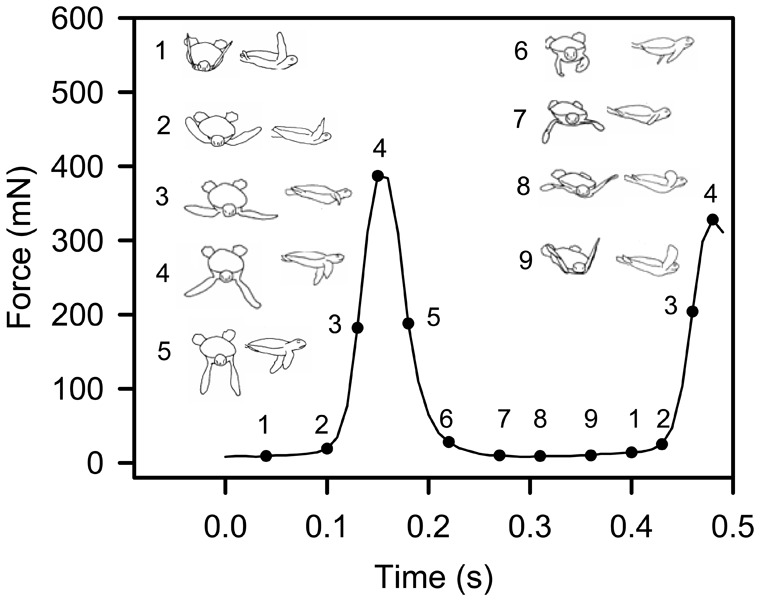
A sample of a force trace illustrating the relative front flipper positions during a typical monophasic stroke cycle of a green turtle hatchling. Hatchling outlines modified from Davenport et al. (Davenport et al., 1984).

Despite the dorsal-ventral movement of front flippers appearing to be similar in all videos, force traces revealed two distinctly different patterns of force production during a front flipper cycle, a monophasic pattern (Fig. 3) and a biphasic pattern (Fig. 4). In the biphasic pattern a small peak in force associated with the end of the upstroke was immediately followed by a much larger peak in force associated with the middle part of the downstroke (Fig. 4, 1 min & 1 h). In all hatchlings that exhibited the biphasic pattern, the pattern was obvious within the first few minutes of entering the water, but became less obvious as swim time progressed, and could appear and disappear within a single power stroking bout (Fig. 2, 1 hr) and ultimately disappeared completely between one and two hours after entering the water. Examination of force production traces from 350 green turtle hatchlings sampled from 42 clutches over three nesting seasons (force traces reviewed from previous studies: Booth, 2009; Ischer et al., 2009; Booth and Evans, 2011; Booth et al., 2013) indicate that 20.6% of hatchlings exhibited this behaviour, and that hatchlings emerging from the same nest at the same time could exhibit either the monophasic or biphasic force production pattern. In hatchlings exhibiting monophasic force production, increasing force was applied during the middle of the downstroke phase (Figs 3, 5).

**Fig. 6.**
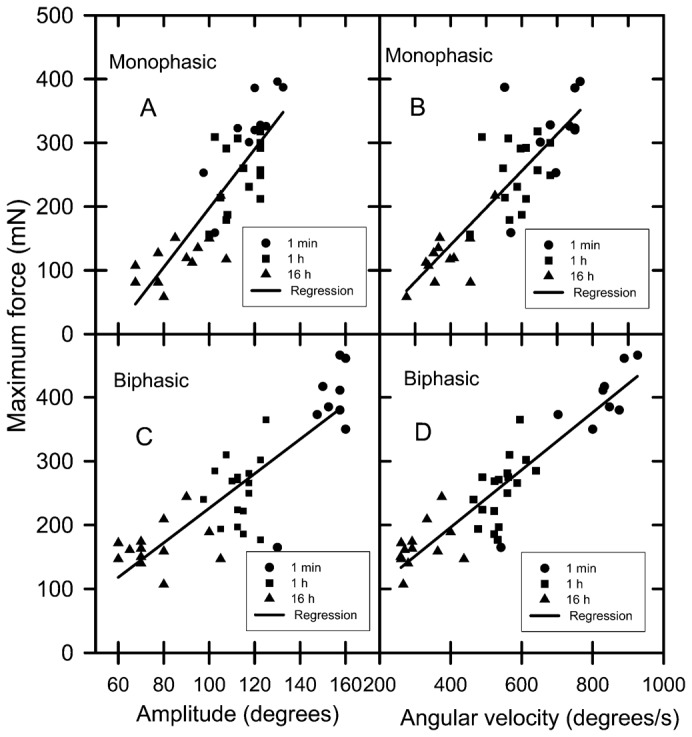
Plots of relationships between front flipper amplitude and peak force and angular velocity and peak force production during the downstroke phase of a powerstroke in green turtle hatchlings. (A) Monophasic flipper amplitude y  =  4.63x − 265, R^2^  =  0.71, n  =  37, P < 0.001. (B) Monophasic flipper angular velocity y  =  0.58x − 91.8, R^2^  =  0.72, n  =  37, P < 0.001. (C) Biphasic flipper amplitude y  =  2.71x − 44.1, R^2^  =  0.71, n  =  40, P < 0.001. (D) Biphasic flipper angular velocity y  =  0.45x + 16.7, R^2^  =  0.72, n  =  40, P < 0.001.

For the hatchling exhibiting monophasic force production, the downstroke time was shorter than the upstroke time during the 1 min and 2 h swimming period, but downstroke and upstroke times were similar during the 16 h swimming period (Table 1). For the hatchling exhibiting biphasic force production, the downstroke time was shorter than the upstroke time during the 1 min swimming period, but downstroke and upstroke times were similar during the 1 h and 16 h swimming periods (Table 1). There was a strong correlation between the force produced during the downstroke and both the amplitude and angular velocity of the flippers during the downstroke in the hatchling exhibiting monophasic and biphasic force production (Fig. 6). Peak force production (Figs 3, 4), flipper amplitude and flipper angular velocity (Fig. 6) decreased as swim time increased.

**Table 1.**
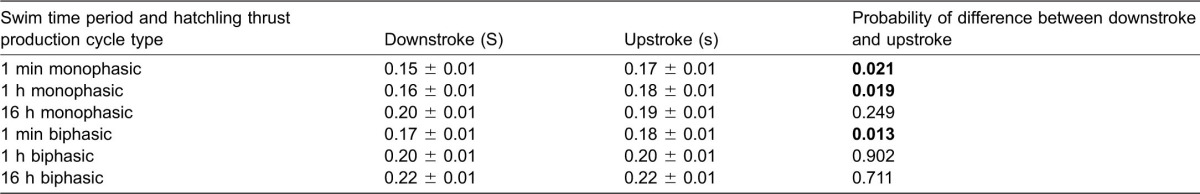
Duration of the downstroke and upstroke phases during a powerstroking bout in tethered swimming green turtle hatchlings. Statistical comparisons made using paired Student's t test

## DISCUSSION

Only the powerstroking aspect of swimming was analysed in detail because it is the most important thrust producing gait and dominates the typical swim frenzy (Wyneken, 1997; Booth, 2009). Without a doubt, the front-flipper movement of green sea turtle hatchlings is extremely complex, and is far from a simple up and down movement (supplementary material Movies 1 and 2). The complex flipper movement has been described previously (Davenport et al., 1984; Wyneken, 1997) and also involves heave, yaw, pitch and roll of the body (Dougherty et al., 2010). The situation is even more complex in newly emerged hatchlings because they are learning to swim for the first time, and have particularly flexible front flippers. A combination of low resolution video and the complex three dimensional movement of both the body and front flippers made the precise tracking of the flipper tips in all video frames impossible so angles of flipper tips were only measured to a accuracy of ± 5°. Despite this limitation, the simple quantification of the vertical up and down movement of the front flippers can reveal valuable insights into swimming kinetics (Davenport et al., 1984; Wyneken, 1997, current study).

### Swim flume experiments

No previous studies have examined the effect of swimming speed on the swimming behaviour of hatchling sea turtles. The range of water speeds tested covered most of the range of swimming speeds estimated for free-swimming green turtle hatchings during the frenzy swim. At the fastest speed tested (0.50 m/s) only half the hatchlings tested were able to swim against the current so this speed approaches the fastest swimming speed possible for the southern great Barrier Reef (GBR) population of green turtle hatchlings. Those that were able to swim at this speed had a stroke rate averaging 180 strokes per minute (SPM), a rate that can only be maintained for less than six minutes in green turtle hatchlings from natural nests (Booth, 2009). However newly emerged green turtle hatchlings from Sabah, Malaysia swam in a flume at speeds averaging 0.63 m/s and were able to maintain this swimming speed for at least 50 min, but no information on hatchling size or powerstroke rate were reported (Pilcher and Enderby, 2001). Clearly there is a difference in the swimming performance of the southern GBR and Sabah populations of green turtle hatchlings and it would be interesting to compare hatchling size, front flipper areas, and stroke rates to see how these can account for their difference in swimming performance.

In the current study, at the slowest water speeds tested (0.20 and 0.25 m/s), hatchlings spent a large proportion of their time dog paddling. Davenport et al. (Davenport et al., 1984) also found juvenile green turtles spent large amounts of time dog paddling at slow speeds. During powerstroking at water speeds up to 0.40 m/s most hatchlings moved forward in the water flume indicating that they were swimming faster than the flume water speed. This makes the relationship between stroke rate during a powerstroking bout and water speed somewhat difficult to interpret. In any case, powerstroke rate was not a good indicator of swimming speed because it was similar at all water speeds tested above 0.25 m/s.

The time taken for the upstroke was slower than the time taken for the downstroke in the vast majority of stroke cycles at all water speeds tested, a finding in agreement with previous studies (Davenport et al., 1984; Rivera et al., 2009). At water speeds greater than 0.20 m/s the duration of upstrokes and downstrokes did not change as water speed increased reflecting the fact that there was no relationship between stroke rate and water speed over this water speed range. Stroke rate was slowest and up and down stroke times longest at 0.20 m/s and an increase in stoke rate can explain an increase in swim speed at this very slow water speed range. However, the finding that the length of time taken for up and down strokes, and as a consequence, the overall stroke rate did not increase with water speeds greater than 0.25 m/s was unexpected because it was assumed that faster swimming would be associated with a faster stroke rate. How can swimming speed increase if stroke rate does not increase? Presumably the swimming speed of a hatchling is directly dependent on the total thrust produced by the flippers, and total thrust production is the product of stroke frequency by thrust produced per stroke. Hence, it follows that because stroke rate is not a good predictor of swimming speed, changes in the thrust produced per stroke must explain the variation in swimming speed at speeds greater than 0.25 m/s because thrust balances drag and drag increases with speed. Differences in thrust per stoke may be caused by differences in stroke amplitude and/or differences in the angle of the flipper blade or even stiffness of the flipper as it moves through the water increased mean thrust coefficient, or increased acceleration reaction due to decreased duration of acceleration phases of stroke cycle. Indeed, there is a relationship between amplitude of flipper movement and maximum thrust force produced (Fig. 6A,C) so changes in flipper amplitude with swim speed is a plausible explanation of how swim speed might increase despite stroke rate remaining constant. In juvenile green turtles stroke frequency did not increase greatly with faster swimming, and this led to the same conclusion i.e. that an increase in swimming speed is achieved mainly by increased front flipper amplitude (Davenport et al., 1984).

Another factor that could lead to increased swim speed while the powerstroking rate within a powerstroking bout remains unchanged is the proportion of time spent powerstroking. At the slowest speed tested (0.20 m/s) hatchling spent only 55% of their time powerstroking, but this increased to 90% at water speeds of 0.45 and 0.50 m/s. The length of the powerstroking bout also increased above water speeds of 0.25 m/s. This indicates that the increase in the proportion of time powerstroking between 0.20 and 0.25 m/s was caused by a shortening of the length of time of dog paddling bouts, but at water speeds of 0.30 m/s and above, further increases in the proportion of time powerstroking were caused by increases in the length of powerstroking bouts.

### Tethered swimming experiments

The hatchlings used in these experiments came from natural nests and were placed into the swim tank within two hours of nest emergence so the swimming effort in the first few minutes can be expected to be maximal (Booth, 2009; Ischer et al., 2009; Booth and Evans, 2011; Booth et al., 2013). Because hatchlings were tethered from above the water surface, the tips of their flippers frequently broke the surface, something that rarely occurs in free-swimming hatchlings. Interestingly, the front flippers breaking the water did not affect the forward thrust force produced during a downward stroke probably because the increase in forward thrust always occurred well into the downstroke phase when the flipper tips were always below the water surface. Even though the smaller force produced during the upstroke phase in the hatchling exhibiting biphasic force production was produced when the flipper tips were close to their uppermost extremity and frequently out of the water, the force produced was not affected by tips of flippers coming out of the water. In this case the lack of effect may be because the region of the flipper that was responsible for producing thrust was located in the proximal region rather than distal region of the flipper, and the proximal region of the flipper was always underwater.

The pattern of front flipper movement during powerstroking observed in the current study was similar to that described for juvenile and hatchling green turtles previously (Davenport et al., 1984; Wyneken, 1997). The monophasic thrust production stroke cycle was the most commonly observed and is reported to only occur in newly emerged hatchlings (Becking et al., 2004). The biphasic stroke cycle was rarely observed in hatchlings in the current study, but is reported as the only stroke style observed in older juveniles (Davenport et al., 1984; Wyneken 1997; Becking et al., 2004). The monophasic hatchling stroke cycle has been described as being simpler, of shorter duration, and less variable than the biphasic stroke cycle used by juveniles (Becking et al., 2004). The exclusive use of the biphasic stroke cycle in hatchlings older than one day suggests that the monophasic stroke cycle observed in new emerged hatchlings is only used during a “learn to swim process” immediately after hatchlings enter the water for the first time. Presumably the biphasic stroke cycle is more efficient at producing forward thrust, but it might take some time to perfect this stroke technique as hatchling need to learn to better coordinate their motor movements. The problem with this “learn to swim process” explanation is that in the current study the use of the biphasic stroke cycle was only ever observed in hatchlings within the first hour or so of entering the water, and that the same individuals switched to the monophasic stroke cycle after this initial swimming period, i.e. the reverse of what would be expected if the biphasic stroke cycle is adopted after an initial learning phase using the monophasic stroke cycle.

The timing of the force changes depicted in Figs 1–3 may be slightly delayed relative to flipper movements because of stretching and contracting of the elastic elements in the lyra harness and nylon tether used in the current experiments. However the timing of maximum force production i.e. towards the end of the downstroke (in both the monophasic and biphasic cycles) and the timing of the small force production in the biphasic cycle (i.e. at the very end of the upstroke) agrees well with the only other study to measured thrust force production and simultaneously record flipper movement in hatchling sea turtles (Becking et al., 2004) and also with observations made on 7–15 month old juvenile green turtles (Davenport et al. 1984).

In the biphasic stroke cycle the force produced during the downstroke was always much greater than the force produced during the upstroke as has been reported previously (Davenport et al., 1984; Becking et al., 2004), and correlates well with the finding that acceleration during the downstroke is greater than the upstroke (Rivera et al., 2009; Rivera et al., 2012). In the literature there is debate on the roles of lift-based and drag-based thrust production during front flipper movement (Davenport et al. 1984; Wyneken 1997; Becking et al., 2004). In reality because of the complex flipper movement which causes the aerodynamic attack angle of the flipper to continuously change, both lift-based and drag-based thrust are likely to be produced, but the relative importance of reach component changes within the stroke cycle. When the attack angle is less than 30° the lift-based mechanism is likely to dominate, and when the angle of attack is greater than 60° the drag-based mechanism is likely to dominate. Image analysis suggests that lift-based thrust should dominate during the early part of the downstroke, and drag-based thrust should dominate during the late part of the downstroke when thrust generation is maximal (Wyneken 1997; Becking et al., 2004). My top view images indicated that the flippers are pulled backward in an anterior to posterior manner within the horizontal plane in a rowing-type motion at the bottom of the downstroke, adding support to the hypothesis that drag-based thrust generation dominates at the time of peak thrust production.

The shallow attach angles during the upstroke suggest that lift-based thrust production is the dominant form of thrust production during the upstroke (Davenport et al., 1984; Wyneken, 1997; Becking et al., 2004). My video images were not clear enough to detect differences in the flipper shape of movement pattern during the upstroke between the monophasic and biphasic stroke cycles so the difference in flipper movement characteristics in these two cycles remain unresolved. In agreement with previous studies, the force production during the upstroke of the biphasic thrust strokes occurred immediately before the end of the upstroke (Davenport et al., 1984; Wyneken, 1997; Becking et al., 2004).

There were differences in thrust force produced by individual strokes within a particular powerstroking bout, and a trend for the thrust per stroke to decreases as the time spent swimming increased. These observations have been noted previously (Booth, 2009; Ischer et al., 2009; Booth and Evans, 2011; Booth et al., 2013) but an explanation of the mechanism for difference in thrust produced per stroke had not been investigated. The relative tight correlation between maximum thrust force produced and the amplitude of the distance moved by flippers during the downstroke suggest that this is the major mechanism by which thrust force is modulated. The amplitude of front flipper movement has also been reported to increase during vigorous compared to routine swimming in juvenile green turtles (Davenport et al., 1984). Other possible mechanisms include subtle changes in flipper attack angle as it moves through the water, but my videos were not of high enough resolution to investigate this aspect.

At the beginning of swimming, the downstroke time interval was typically faster than the up stroke interval as has been reported previously (Davenport et al., 1984; Rivera et al., 2009; and swim flume experiments reported above) but after 16 h of swimming the upstroke and downstroke intervals were similar. This relatively slower downstroke time probably reflect the facts that the hatchlings are more fatigued and the downstroke is when thrust is produced and thus requires more muscular effort than the upstroke.

### Conclusion

The front flippers and body are involved in complex three-dimensional movements during powerstroking in green turtle hatchlings. Despite this complexity, information from simple analysis of stroke timing and stroke amplitude provided valuable information on thrust production during powerstroking bouts. Although the video image analysis was unable to distinguish between them, two types of powerstroke cycle were identified from thrust traces, a common monophasic and a rarer biphasic thrust cycle. During monophasic thrust production, thrust was generated during the downstroke and reached peak thrust midway to three-quarters of the way through the downstroke. Thrust generated early in the downstroke was most likely generated by lift-based thrust while drag-based thrust was most likely responsible for the peak in thrust production that occurred later in the downstroke. Peak thrust during the biphasic thrust cycle occurred during the downstroke and had similar characteristics to the monophasic cycle, while the smaller peak in thrust occurred at the very end of the upstroke and most likely was generated by lift-based thrust. Unexpectedly, swimming speed was not correlated with stroke rate, but instead appeared to be a function of stroke amplitude and proportion of time spent powerstroking.

## MATERIALS AND METHODS

### Swimming flume experiments

Four clutches of green turtle eggs were collected immediately after oviposition from females on Heron Island, (23°26′S, 151°51′E), Southern Great Barrier reef (GBR) in December 2013 and transported to a laboratory at the University of Queensland in Brisbane where eggs were incubated at a constant temperature of 28°C buried in vermiculite with a water potential of ∼100 kPa. Hatchlings were maintained in the incubation container for 48 h after they hatched to mimic the time they would spend digging out of the nest in nature, and then allowed to crawl inside a bucket for between one and two hours before being swum. Hatchlings swum unimpeded in a Loligo systems (Tjele, Denmark) 185 L swim tunnel with a 85 cm L × 25 cm W × 25 cm H working section filled with freshwater heated to 28°C. Water speed within the working section was maintained with a precision controller, and measured with a flow sensor model HA connected to a hand held model HFA Flow meter (Hontzsch: Waiblingen, Germany). Swimming within the flume was videoed at 300 frames per second with a Casio EX-F1digital camera (Tokyo, Japan) mounted 0.5 m from the side and centre of the working section at working section height. Hatchlings were placed in the flume with water speed initially set at 0.20 m/s for two minutes, and when videoed for 1 minute. The water speed was then stepped up sequentially to 0.25, 0.30, 0.35, 0.40, 0.45 and 0.50 m/s and hatchlings acclimated to the new speed for one minute before being videoed for one minute. Not all hatchlings could sustain swimming at higher water speeds so they were removed from the flume and not tested at higher speeds.

Front flipper movement was only analysed during powerstroking bouts, dog paddling bouts were ignored. During data extraction videos were played back frame by frame using Apple QuickTime Player (Ver 7.7.5) on a flat screen monitor. For each hatchling, at each water speed, five separate power stroking bouts were analysed. For each of these bouts the frame number at the beginning of a bout was subtracted from the frame number at the end of the bout and this number divided into 300 to calculate the bout length in seconds. The number of strokes taken during the bout was multiplied by 60 and divided by the bout length (s) to calculate the power stroking rate (SPM) for that bout. Within each bout the frame number at which the front flippers reached their maximum upward and downward sweep was recorded, and the number of frames between each of these extreme flipper positions was used to calculate the time taken for the up-stroke and down-stroke.

### Tethered hatchling experiments

**Fig. 7.**
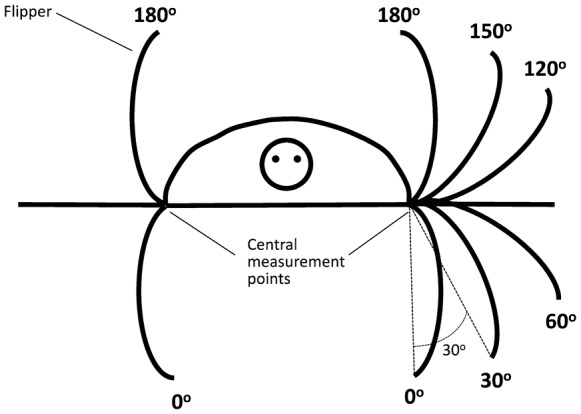
Schematic diagram illustrating how the front flipper tip angle was measured during up and down flipper movement within a powerstroking bout.

Experiments were performed on 10 green turtle hatchlings collected from beach nests on Heron Island between 25 January and 10 February 2009. Hatchlings were collected within an hour of emerging onto the beach surface by placing enclosures above nests that were due to emerge and transferred in a bucket on foot to the laboratory (maximum of 15 minutes). Hatchlings crawled around within the enclosure and bucket, a behaviour that mimicked that of a hatchling crawling from the nest to the sea. Once in the laboratory hatchlings were fitted with lycra harnesses which did not inhibit flipper movement. They swum in a plexiglass chamber (34 cm long × 28 cm wide × 19 cm high) filled to 13 cm with fresh seawater heated to 28°C by an aquarium heater. The harness was attached via a monofilament nylon line, which passed through an eyelet mounted on a beam fixed to the top of the tank and was attached to a force transducer (MLT050 ADInstruments: Colorado Springs, USA) connected to a bridge amplifier (ML112 ADInstruments: Colorado Springs, USA). The output was recorded via a data acquisition system (Power Lab 4/20 ADInstruments: Colorado Springs, USA) programmed to sample 100 times per second. Before and after each trial, the force transducer was calibrated by hanging a known mass from it. A mini florescent light was placed in front of the tank to encourage uni-directional swimming. Mirrors angled at 45° were place above the tank and to one side so that swimming could be viewed in three dimensions (supplementary material Movie 2). Swimming was videoed at 300 frames per second for between 1 and 2 minutes at various times after hatchlings were placed in tanks with a Casio EX-F1digital camera (Tokyo, Japan). A red LED was wired to the powerlab and placed to the side of the tank and triggered to fire once per second for five seconds during each video to enable synchronization of the video frames with the sampling time of the force transducer. Front flipper movement was only analysed during powerstroking bouts, dog-paddling bouts were ignored. During data extraction videos were played make frame by frame using Apple QuickTime Player (Ver 7.7.5) on a flat screen monitor. The flashes were identified and used to align the frame number with the time record of the chart file that had recorded the output of the force transducer. The video was advanced 3 frames at a time (which corresponded to the 0.01 second sampling interval of the force data) and the position of the tip of the left and right front flippers from the front-on image of the hatchling measured with a protractor to the nearest 5°, with zero degrees corresponding to vertically below the joint of the flipper and the body and 180° corresponding to vertically above the joint of the flipper and the body (Fig. 7). Angular velocity during the downstroke of the front flippers was calculated by dividing the angle that the flippers transversed during the downstroke by the time taken for the downstroke.

### Statistical analysis

Linear regression was used to investigate the relationship between front flipper amplitude and force produced per stroke and front flipper angular velocity and force produced per stroke in tethered swimming experiments. Paired Student's t test was used to compare the time taken for an upstroke and downstroke in both tethered swimming and flume swimming experiments. Repeat measures ANOVA was used to test for differences in power stroke rate, time taken for downstroke, time taken for up stroke, and time difference between up stroke and down stroke across the different water speeds in swim flume experiments. Where ANOVA indicated a significant difference the Tukey procedure was used to compare different water speeds. The program Statistica Ver 12 was used for statistical analysis, and statistical significance assumed if α < 0.05.

## Abbreviations

Carapace length (CL), carapace width (CW), Great Barrier Reef (GBR), stroke per minute (SPM).

## Supplementary Material

Supplementary Material

## References

[b1] Becking L. E. Blob R. Wyneken J. (2004). Three-dimensional kinematic analysis of powerstroking by hatchling and pelagic stage loggerhead sea turtles Caretta caretta L. *J. Morphol.* 260, 277.

[b2] Booth D. T. (2009). Swimming for your life: locomotor effort and oxygen consumption during the green turtle (Chelonia mydas) hatchling frenzy. J. Exp. Biol. 212, 50–55 10.1242/jeb.019778 19088210

[b3] Booth D. T. Evans A. (2011). Warm water and cool nests are best. How global warming might influence hatchling green turtle swimming performance. PLoS ONE 6, e23162 10.1371/journal.pone.0023162 21826236 PMC3149641

[b4] Booth D. T. Feeney R. Shibata Y. (2013). Nest and maternal origin can influence morphology and locomotor performance of hatchling green turtles (Chelonia mydas) incubated in field nests. Mar. Biol. 160, 127–137 10.1007/s00227-012-2070-y

[b5] Carr A. F. Ogren L. (1960). The ecology and migration of sea turtles. 4. The green turtle in the Caribbean Sea. Bull. Am. Mus. Nat. Hist. 121, 6–48.

[b7] Davenport J. Munks S. A. Oxford P. J. (1984). A comparison of the swimming of marine and freshwater turtles. Proc. R. Soc. B 220, 447–475 10.1098/rspb.1984.0013

[b6] Dougherty E. Rivera G. Blob R. Wyneken J. (2010). Hydrodynamic stability in posthatchling loggerhead (Caretta caretta) and green (Chelonia mydas) sea turtles. Zoology 113, 158–167 10.1016/j.zool.2009.10.001 20417080

[b8] Frick J. (1976). Orientation and behaviour of hatchling green turtles (Chelonia mydas) in sea. Anim. Behav. 24, 849–857 10.1016/S0003-3472(76)80015-2

[b9] Gyuris E. (1994). The rate of predation by fishes on hatchlings of the green turtle (Chelonia mydas). Coral Reefs 13, 137–144 10.1007/BF00301189

[b10] Gyuris E. (2000). The relationship between body size and predation rates on hatchlings of the green turtle (Chelonia mydas): is bigger better? Sea Turtles of the Indo-Pacific: Research, Management and Conservation Pilcher N J Ismail M G, ed143–147 New York, NY: Academic Press.

[b11] Ischer T. Ireland K. Booth D. T. (2009). Locomotion performance of green turtle hatchlings from the Heron Island rookery, Great Barrier Reef. Mar. Biol. 156, 1399–1409 10.1007/s00227-009-1180-7

[b12] Okuyama J. Abe O. Nishizawa H. Kobayashi M. Yoseda K. Arai N. (2009). Ontogeny of the dispersal migration of green turtle (Chelonia mydas) hatchlings. J. Exp. Mar. Biol. Ecol. 379, 43–50 10.1016/j.jembe.2009.08.008

[b13] Pereira C. M. Booth D. T. Limpus C. J. (2011). Locomotor activity during the frenzy swim: analysing early swimming behaviour in hatchling sea turtles. J. Exp. Biol. 214, 3972–3976 10.1242/jeb.061747 22071188

[b14] Pilcher N. J. Enderby S. (2001). Effects of prolonged retention in hatcheries on green turtle (Chelonia mydas) hatchling swimming speed and survival. J. Herpetol. 35, 633–638 10.2307/1565902

[b15] Pilcher N. J. Enderby S. Stringell T. Bateman L. (2000). Nearshore turtle hatchling distribution and predation. Sea Turtles of the Indo-Pacific: Research, Management and Conservation Pilcher N J Ismail M G, ed151–166 New York, NY: Academic Press.

[b16] Rivera A. R. V. Bennett N. L. Rivera G. Wyneken J. Blob R. W. (2009). Whole-body acceleration during swimming in the green sea turtle (Chelonia mydas): a comparison of upstroke and downstroke. Integr. Comp. Biol. 49, E297.

[b17] Rivera A. R. V. Wyneken J. Blob R. W. (2011). Forelimb kinematics and motor patterns of swimming loggerhead sea turtles (Caretta caretta): are motor patterns conserved in the evolution of new locomotor strategies? J. Exp. Biol. 214, 3314–3323 10.1242/jeb.057364 21900480

[b18] Rivera A. R. V. Rivera G. Blob R. W. Wyneken J. (2012). Whole-body acceleration and inertial effects of flippers during swimming in the green sea turtle (Chelonia mydas). Integr. Comp. Biol. 52, E319.

[b19] Salmon M. Wyneken J. (1987). Orientation and swimming behavior of hatchling loggerhead turtles Caretta caretta L. during their offshore migration. J. Exp. Mar. Biol. Ecol. 109, 137–153 10.1016/0022-0981(87)90012-8

[b20] Salmon M. Hamann M. Wyneken J. Schauble C. (2009). Early swimming activity of hatchling flatback sea turtles Natator depressus: a test of the ‘predation risk’ hypothesis. Endanger. Species Res. 9, 41–47 10.3354/esr00233

[b21] Spotila J. A. (2004). Sea Turtles: A Complete Guide to Their Biology, Behavior, and Conservation Baltimore, MD: Johns Hopkins University Press.

[b22] Whelan C. L. Wyneken J. (2007). Estimating predation levels and site-specific survival of hatchling loggerhead sea turtles (Caretta caretta) from South Florida beaches. Copeia 2007, 745–754 10.1643/0045-8511(2007)2007[745:EPLASS]2.0.CO;2

[b23] Wyneken J. (1997). Sea turtle locomotion: mechanisms, behavior, and energetics. Biology of Sea Turtles Lutz P, ed165–198 New York, NY: CRC Press.

[b24] Wyneken J. Salmon M. (1992). Frenzy and postfrenzy swimming activity in loggerhead, green, and leatherback hatchling sea turtles. Copeia 1992, 478–484 10.2307/1446208

