# Supplementary material for: Kinematics of swimming and thrust production during powerstroking bouts of the swim frenzy in green turtle hatchlings
Source: Biol Open. 2014 Sep 4;3(10):887–94. doi: 10.1242/bio.20149480 (PMC4197437; doi:10.1242/bio.20149480)
Supplement: Supplementary Material [file supp_3_10_887__index.html]

Kinematics of swimming and thrust production during powerstroking bouts of the swim frenzy in green turtle hatchlings — Supplementary Material 

# Kinematics of swimming and thrust production during powerstroking bouts of the swim frenzy in green turtle hatchlings

## bio.20149480 Supplementary Material

**Files in this Data Supplement:**

- Supplementary Material - David T. Booth doi: 10.1242/bio.20149480
- Movie 1 - **Movie 1. A sample video of a hatchling swimming in the swimming flume at a water speed of 0.5m/s.**
- Movie 2 - **Movie 2. A sample of video footage of powerstroking taken during tethered hatchling swimming experiments. This video was taken within the first minute of the hatchling being placed in water, and the hatchling's force trace was monophasic.**
