# Supplementary material for: Kinematics of swimming and thrust production during powerstroking bouts of the swim frenzy in green turtle hatchlings
Source: Biol Open. 2014 Sep 4;3(10):887–94. doi: 10.1242/bio.20149480 (PMC4197437; doi:10.1242/bio.20149480)
Supplement: Supplementary Material [file supp_bio.20149480_bio.20149480-s1.pdf]

Biology Open (2014) 000, 1–8 doi:10.1242/bio.20149480

Supplementary Material

David T. Booth doi: 10.1242/bio.20149480

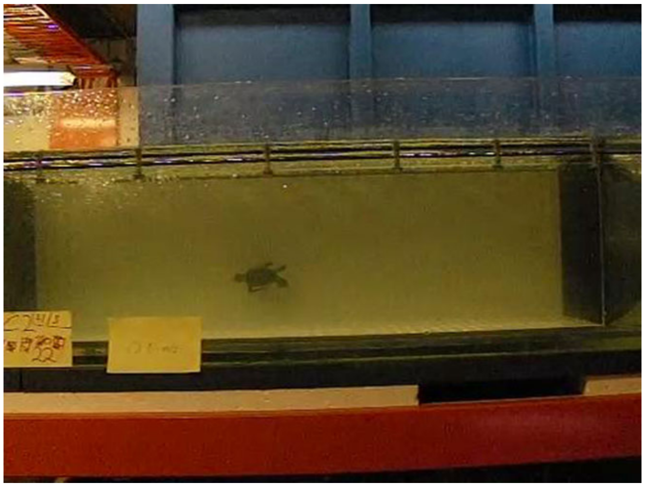

Movie 1. A sample video of a hatchling swimming in the swimming flume at a water speed of 0.5m/s.

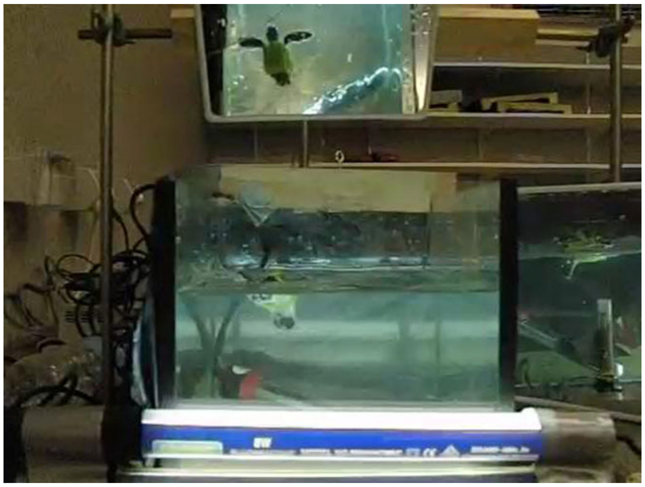

Movie 2. A sample of video footage of powerstroking taken during tethered hatchling swimming experiments. This video was taken within the first minute of the hatchling being placed in water, and the hatchling's force trace was monophasic.

Biology Open
